# Progress and opportunities in bismuth-based materials for X-ray detection

**DOI:** 10.1557/s43581-025-00142-5

**Published:** 2025-08-25

**Authors:** Joydip Ghosh, Priyanka Priyadarshini, Zubaida T. Younus, Quanxi Jia, Wanyi Nie, Judith L. MacManus-Driscoll, Robert L. Z. Hoye

**Affiliations:** 1 Inorganic Chemistry Laboratory, University of Oxford, Oxford, OX1 3QR UK; 2 Department of Materials Science and Metallurgy, University of Cambridge, Cambridge, CB3 0FS UK; 3 Department of Materials Design and Innovation, University at Buffalo - The State University of New York, Buffalo, NY 14260 USA; 4 Department of Physics, University at Buffalo - The State University of New York, Buffalo, NY 14260 USA

**Keywords:** Bi, perovskites, electronic material, devices, defects, electron–phonon interactions

## Abstract

Over the past decade, lead halide perovskites have gained significant interest for ionizing radiation detection, owing to their exceptional performance, and cost-effective fabrication in a wide range of form factors, from thick films to large single crystals. However, the toxicity of lead, limited environmental and thermal stability of these materials, as well as dark current drift due to ionic conductivity, have prompted the development of alternative materials that can address these challenges. Bismuth-based compounds (including perovskite derivatives and nonperovskite materials) have similarly high atomic numbers, leading to strong X-ray attenuation, but have lower toxicity, tend to be more environmentally stable, and can have lower ionic conductivity, especially in low-dimensional materials. These materials are also advantageous over commercial direct X-ray detectors by being able to detect lower dose rates of X-rays than amorphous selenium by at least two orders of magnitude, are potentially more cost-effective to mass produce than cadmium zinc telluride, and can operate at room temperature (unlike high-purity Ge). Given the strong interest in this area, we here discuss recent advances in the development of bismuth-based perovskite derivatives (with 3D, 2D and 0D structural dimensionality), and other bismuth-based perovskite-inspired materials for direct X-ray detection. We discuss the critical properties of these materials that underpin the strong performances achieved, particularly the ability to detect low-dose rates of X-rays. We cover key strategies for enhancing the performance of these materials, as well as the challenges that need to be overcome to commercialize these emerging technologies.

**Graphical abstract:**

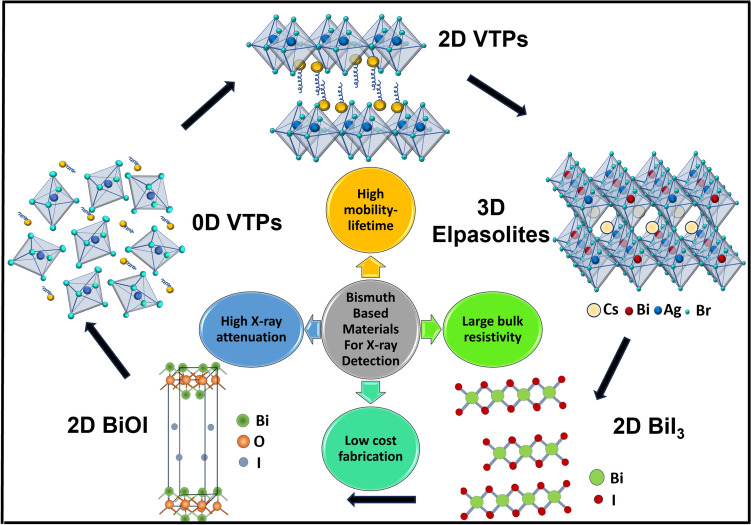

## Highlights

Lead-free bismuth-based perovskites and derivatives are promising eco-friendly materials for sensitive X-ray detection, crucial for medical imaging and security inspection applications. This review highlights their key properties, recent developments, strategies to enhance performance, as well as commercialization challenges.

## Discussion

- Material upscaling is an ongoing effort for Bi-based materials to enable large-area radiation imaging.
- Approaches to control charge-carrier transport and localization are not well understood in the Bi-based material systems, and it will be important to develop materials design principles to improve charge-carrier transport.
- The chemistry and physics of defects in Bi-based semiconductors are not well understood, and addressing defect-mediated non-radiative recombination would greatly improve performance.

## Introduction

X-rays are high-energy photons in the high-frequency part of the electromagnetic spectrum, and are capable of penetrating through solids and soft tissue. As a result, X-ray detection is widely used for non-destructive measurements across a variety of disciplines, including medical imaging, security screening, and in-field inspection.^1,2^

The popular approaches for X-ray detection are based on scintillators and solid-state devices for indirect and direct conversion of the X-ray photon signal to an electrical signal, respectively. A scintillator is comprised luminescent materials that can downconvert high-energy X-ray photons to lower-energy visible photons. It is typically coupled to a visible light detector, such as an avalanche photodiode or a photon multiplication tube. This is also called an indirect detector because of the multiple conversion processes involved (X-rays to visible photons, visible photons to an electrical signal). Direct detectors, on the other hand, utilize semiconductors to convert X-ray photons to electrical signals. Semiconductors commonly used commercially for direct conversion include silicon, germanium, GaAs, Cd-Zn-Te and amorphous selenium (α-Se).^3^ Directly converting X-ray photons to an electrical signal can lead to higher conversion efficiencies, especially in the low-dose range, as well as higher spatial resolution compared to scintillators.^4^

The types of detectors employed depend on the specific end uses. For instance, for experiments requiring fast time resolution, scintillators are typically chosen for their rapid radioluminescence decay on the sub-nanosecond timescale.^5^ The response time of the direct conversion detector is determined by the charge extraction time, which is typically longer than 10 s of nanoseconds. In the case of medical imaging, where the main consideration is minimizing the patient’s X-ray exposure dose, it is more important to have a highly sensitive detector with a strong signal-to-noise ratio (SNR) in a low flux regime. In this scenario, a direct detection mechanism with a high detection quantum efficiency is preferred. In imaging applications, spatial resolution is another important figure-of-merit to consider when choosing detector materials. Here, direct detectors are advantageous, in that the pixels used can be made as small as 10 s of microns, enabling high spatial resolution to be achieved. For radiology, a resolution of 5.7 lp mm^–1^ is required, while mammography demands a higher resolution, with a minimum requirement of 10 lp mm^–1^.^6,7^ However, there are currently few direct detector materials in the market right now. One limitation is the high cost of building digital panels using classical semiconductors, such as cadmium zinc telluride (CZT).

Recently, remarkable developments have been made in low-cost lead halide perovskites (LHPs) for direct radiation detection thanks to the ability to achieve high mobility-lifetime products using simple solution-based synthesis methods, along with high X-ray attenuation coefficients, large bulk resistivities, and high radiation hardness.^7–11^ Solution-grown LHP single crystals (SCs) have demonstrated high sensitivities, and the capability of detecting remarkably low-dose rates of X-rays.^11^ Organic–inorganic hybrid perovskite direct X-ray detectors have achieved a record X-ray sensitivity of ~ 2.2 × 10^8^ µC Gy_air_^−1^ cm^−2^, along with a low detection limit of 0.62 nGy_air_ s^−1^.^12–14^ By contrast, industry-standard amorphous selenium direct detectors have sensitivities of 20 μC Gy_air_^−1^ cm^−2^ and the lowest detectable dose rate of 5500 nGy_air_ s^−1^.^15^ However, the commercial application of LHP-based devices is mainly restricted by poor ambient stability. LHP devices degrade in the presence of oxygen, moisture, high temperatures, and extreme light.^16^ Another shortcoming of LHP radiation detectors is the toxicity of lead, which is bioaccumulative, and can readily be released from LHPs owing to their water solubility. Toxic solvents (*e.g.*, *N*,*N*-dimethylformamide or chlorobenzene) are often used in the synthesis of LHPs, and the ambient stability of these materials is limited, such that their commercial use depends on effective encapsulation. The exceptional performance of LHPs, as well as their limitations, have prompted a broad search for more sustainable alternatives, and Bi-based materials have emerged as a prominent class of materials.

In this review, we discuss the status, challenges and future opportunities of using Bi-based perovskite-inspired materials for direct X-ray detection. We begin with the key requirements of X-ray detectors, the properties of Bi-based materials that allow them to fulfill these properties, and the progress in applying these materials in devices. We conclude with a discussion of the market opportunities for these materials, and the challenges in commercializing these devices.

## Key properties of X-ray detector materials

Achieving high-resolution X-ray imaging with minimal radiation exposure to the subject (*e.g.*, medical patient) requires an in-depth understanding of the interplay between materials properties and device performance. For instance, the X-ray attenuation coefficient, ionization energy, and mobility-lifetime product are closely related to the chemistry, microstructure, and defect structure of the materials.^17^ These also affect important device properties, such as dark current, charge collection efficiency, sensitivity, detection limit, and response time.^18,19^ In the following, we will discuss some key properties and figures of merit for radiation detector materials.

### Radiation attenuation

The attenuation of radiation in materials typically occurs through Rayleigh scattering, photoelectric effects, Compton scattering, and pair production. X-ray attenuation is quantified by the Beer-Lambert law:

1 $$I = I_{0} \exp \left\{ { - \mu_{MAC} \rho_{MD} x} \right\}$$

where *I* is the attenuated radiation intensity at a given depth of *x* from the surface exposed to X-rays, *I*_0_ is the incident radiation intensity at *x* = 0, and μ_MAC_ and ρ_MD_ are the mass attenuation coefficient and the mass density of the material, respectively. The value of μ_MAC_ is proportional to *Z*^4^, where *Z* is the average atomic number of the material. Therefore, effective radiation detection requires materials with high atomic numbers (*Z* > 40) and mass density to ensure that sufficient X-ray attenuation can be achieved without requiring excessively large thicknesses that are challenging for charge-carrier extraction. For instance, it has been reported that the effective *Z* (*Z*_eff_) value is 73.6 for BiOI. Such a material could lead to a stopping power nearly double that of CZT (*Z*_eff_ = 48–52) at energies > 100 keV, and is also substantially higher than amorphous Se (*Z*_eff_ = 34).^2,20^

### Charge collection efficiency

The charge collection efficiency (CCE) is the ratio of the total charge collected to the charge generated within a material when the device is exposed to radiation. CCE reflects how effectively a detector converts incoming radiation into a measurable electrical signal. As depicted by Eq. (2), the mobility-lifetime product ($\mu \tau$) of charge carriers plays an important role in determining the CCE at a given generation rate of electron–hole carriers, where $\mu$ and $\tau$ are the carrier mobility and lifetime, respectively. A large $\mu \tau$ product will ensure that generated charge carriers can be collected before they are lost due to non-recombination.

2 $$CCE = \frac{\mu \tau V}{{L^{2} }}\left[ {1 - \exp \left( { - \frac{{L^{2} }}{\mu \tau V}} \right)} \right]$$

### Sensitivity

Sensitivity (*S*) is defined by the charge accumulated per unit area when the device is exposed to radiation. This performance metric reflects a material’s efficiency in converting irradiated photons into electrical signals. Mathematically, *S* can be expressed as

3 $$S = \frac{{I_{radiation} - I_{dark} }}{DA}$$

where *D* is the irradiation dose rate, *A* is the active area of the detector, and *I*_radiation_ and *I*_dark_ are the currents from the device with and without irradiation, respectively. To enhance *S* for a given device design, it is critical to reduce *I*_dark_, as well as to increase CCE to increase the photocurrent signal. Reductions in *I*_dark_ could be achieved by increasing the bandgap of the material (thus lowering the concentration of thermally generated charge-carriers), lowering the doping level (*e.g.*, by reducing the concentration of donor/acceptor defects), or by lowering the electronic dimensionality of the material. For example, many efforts have been made to synthesize single crystals (SCs) or large-grained polycrystalline materials in order to improve $\mu \tau$ products (by reducing grain boundary scattering of charge-carriers, as well as reducing non-radiative recombination), and lower dark currents due to point defects.^21^ It is worth noting that increases in the measured sensitivity can also be achieved from photoconductive gain, which could be detrimental if it also increases the noise current, and increases the detection limit.^22^

### Signal-to-noise ratio

The SNR is a measure of the device’s ability to produce a detectable signal. Using current as the detected signal, the SNR can be expressed as^23,24^

4 $$SNR = \frac{{I_{SC} }}{{I_{NC} }}$$

where *I*_*SC*_ and *I*_*NC*_ are the signal current and noise current, respectively, where *I*_SC_ is taken as the photocurrent (*I*_*radiation*_—*I*_*dark*_). The *I*_*NC*_ can, however, come from different sources. For example, the Johnson noise current and the current resulting from the limited shunt resistance of the device can all contribute to *I*_NC_.

### Limit of detection

In addition to sensitivity, another key metric for X-ray detectors is the lowest detectable dose rate (LoDD), where having a low LoDD is critical for minimizing harm to the patient during medical imaging.^24,25^ The LoDD for radiation detectors is defined by IUPAC as the radiation dose rate at which the SNR of the detector is 3. The SNR is typically calculated as the ratio of the average photocurrent signal to the standard deviation of the photocurrent, while the LoDD is determined by measuring the SNR across various dose rates.

## Key properties of bismuth-based materials for X-ray detection

Bismuth-based materials have emerged as a promising class of compounds for X-ray detection because of their low toxicity, strong attenuation of X-rays, and, in many cases, high environmental and thermal stability.^26^ Bismuth is the heaviest (*Z* = 83) element that is not radioactive, and the high effective atomic number of Bi-based compounds is critical for their high mass attenuation coefficients for ionizing radiation (Sect. “Radiation attenuation”). Some Bi-based materials have also exhibited high mobility-lifetime products (> 10^–4^ cm^2^ V^–1^), along with bandgaps that can be tuned to the optimal range for X-ray detectors (1.4–2.5 eV).^7,27^ Prominent classes of Bi-based materials are halide elpasolites (A_2_M^I^M^II^X_6_, e.g., Cs_2_AgBiBr_6_), vacancy-ordered triple perovskites (A_3_Bi_2_X_9_; (NH_4_)_3_Bi_2_I_9,_ MA_3_Bi_2_I_9_, Cs_3_Bi_2_I_9_), and other binary or mixed-anion bismuth-based compounds (BiOI, BiI_3_, Bi_2_O_3_).

### Bismuth-based double perovskites

**Figure 1:**
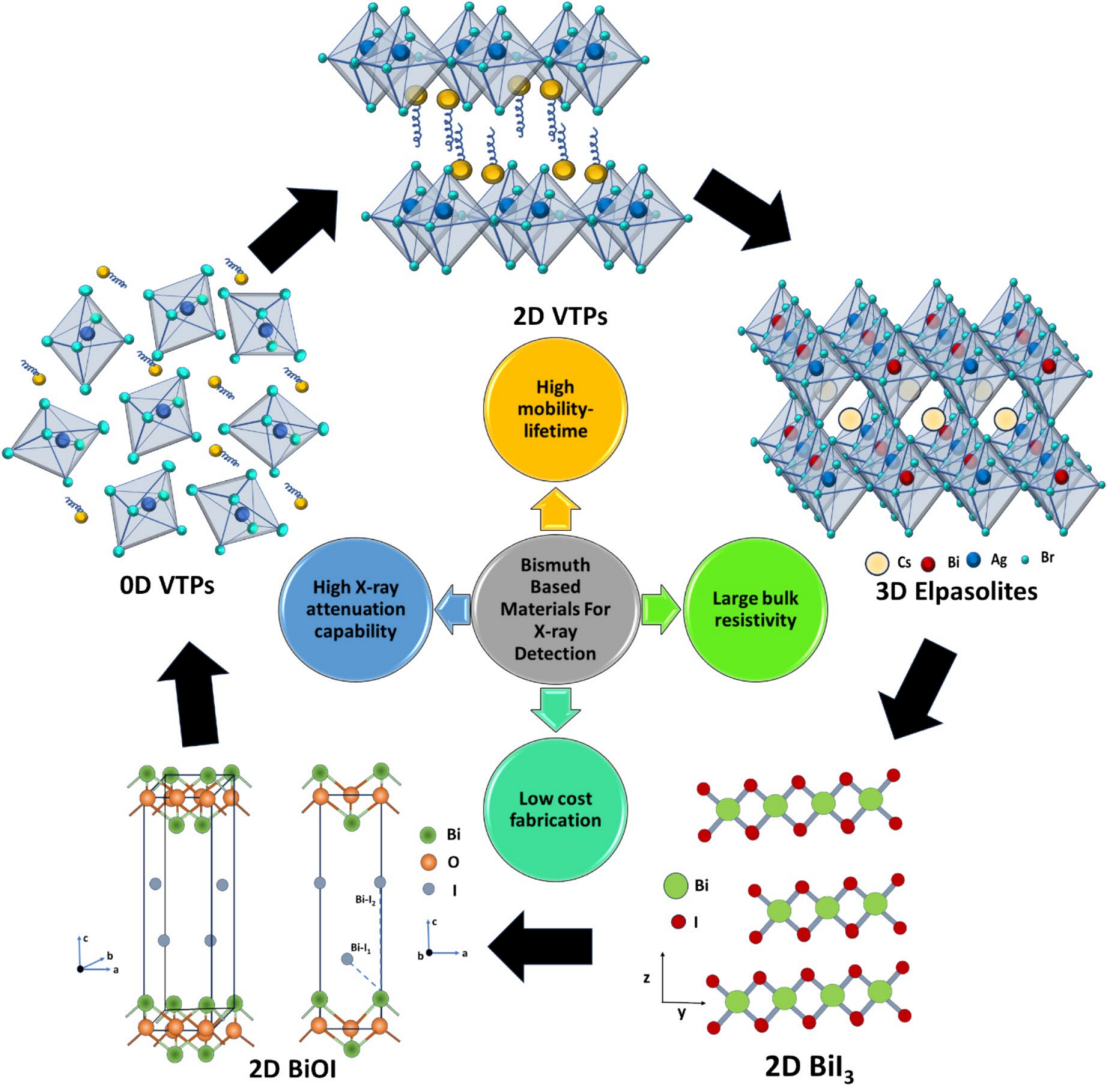
Schematic representation of different key properties of bismuth-based materials for X-ray detection.

**Figure 2:**
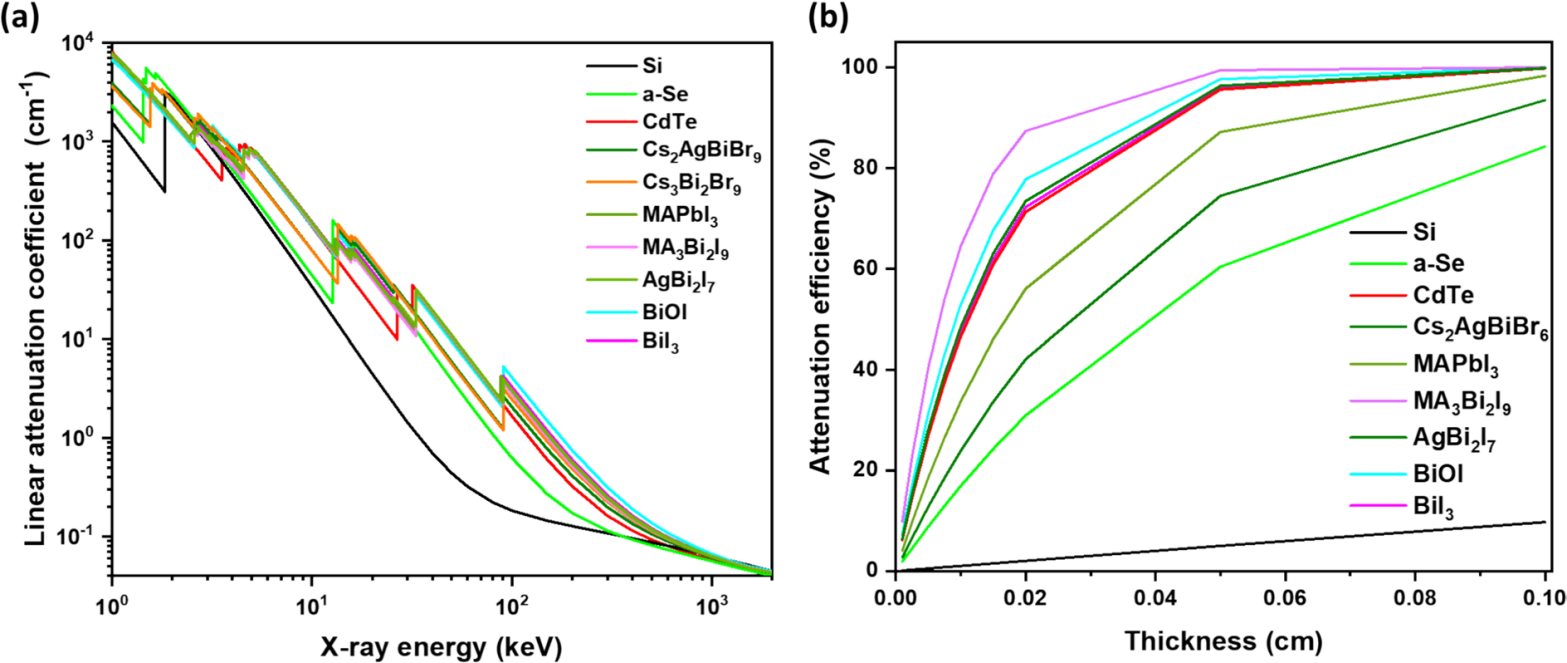
Comparison of the linear attenuation coefficient versus X-ray energy of different bismuth-based materials with conventional lead-halide perovskites and semiconductors used commercially in X-ray detectors. (b) Attenuation efficiency versus thickness of bismuth-based materials compared with conventional semiconductors. Calculations were made based on 50 keV X-ray photon irradiation. Data for Fig (a) are obtained from the NIST database,^34^ while part (b) is calculated using the data shown in part (a) and using Eq. (1).

**Figure 3:**
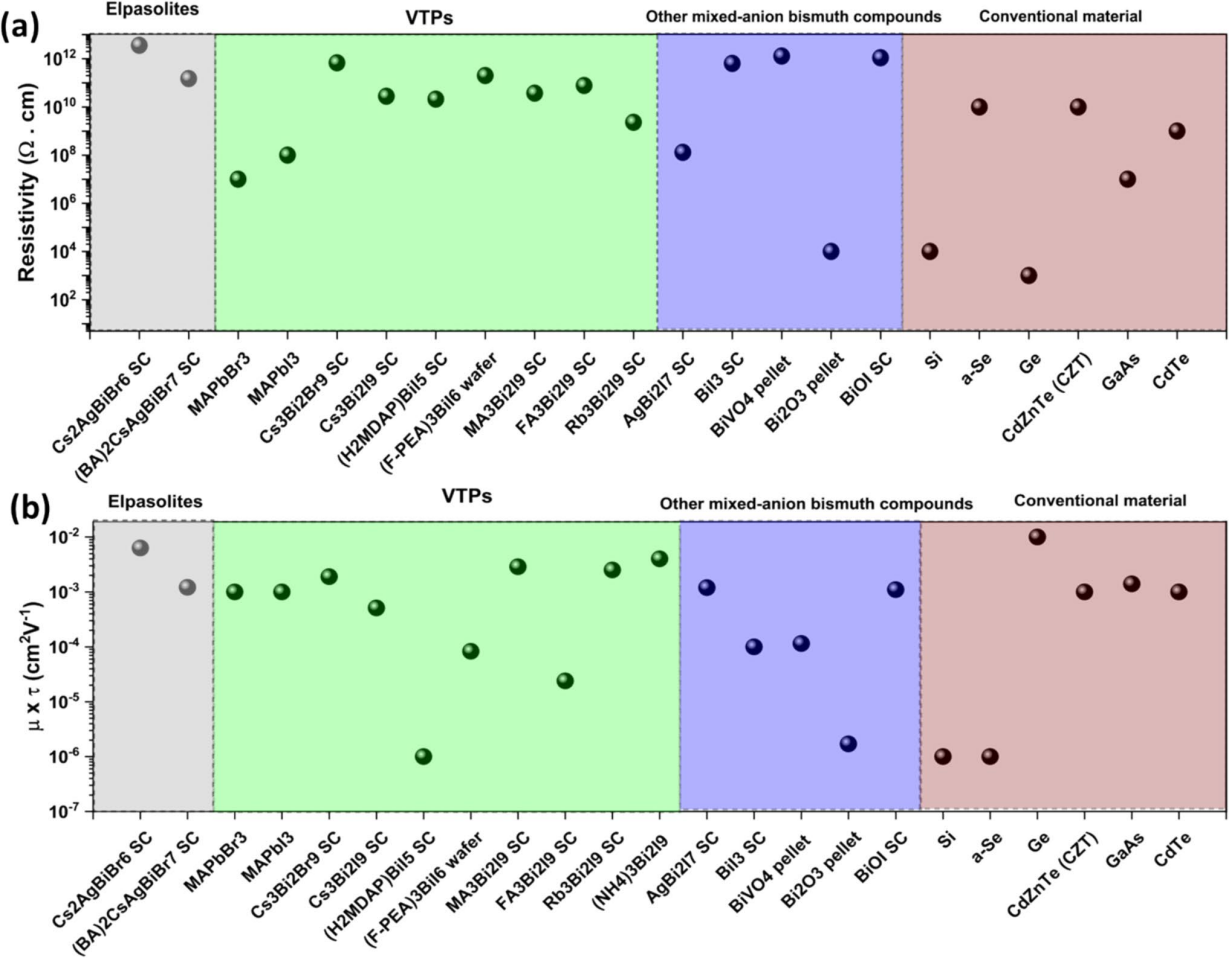
Comparison of (a) resistivity, and (b) mobility-lifetime product of some of the best performing Bi-based materials, compared with conventional state-of-art semiconductors used for X-ray detection. The μτ product of Bi-based materials showed slight lower value than MAPbI_3_, but exceeds most semiconductors used commercially in X-ray detectors. Data collected from the references in Table 1. SC refers to single-crystal material.

**Figure 4:**
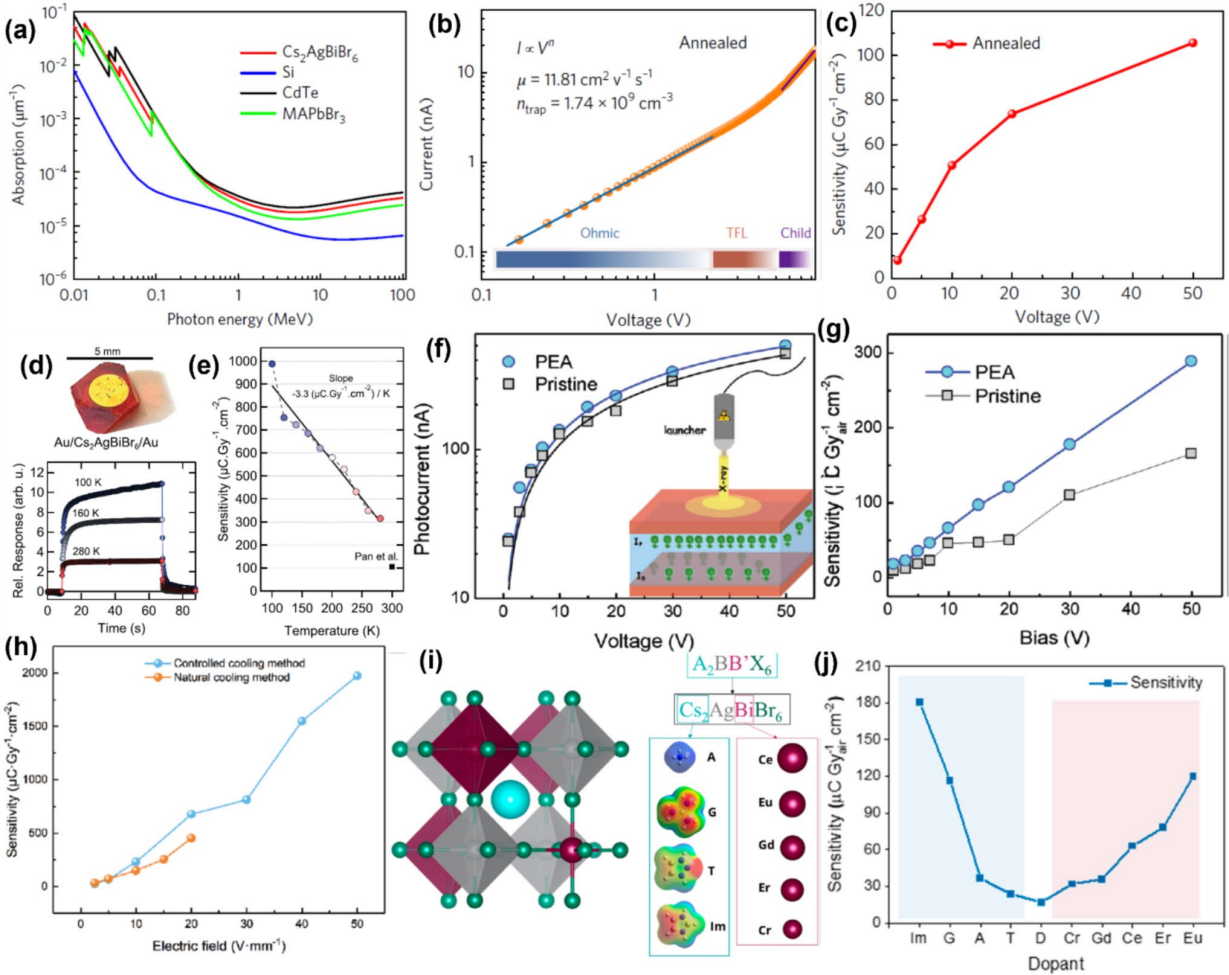
(a) Comparison of the attenuation coefficients of Cs_2_AgBiBr_6_ double perovskite with MAPbBr_3_, CdTe, and Si as a function of photon energy. (b) Current–voltage characteristics of Cs_2_AgBiBr_6_ single crystal, featuring linear and quadratic fittings based on the space charge-limited current (SCLC) model. (c) The sensitivity of the SC detector under different bias voltages. Reproduced from, Ref. 28 with permission from Springer Nature. (d) Top: photograph of Au/Cs_2_AgBiBr_6_/Au X-ray detector, bottom: Temporal X-ray current of the detector at different measurement temperatures and applied electric field of 50 V mm^−1^. (e) X-ray sensitivities of the device at different measurement temperatures. Reproduced from, Ref. 44 John Wiley & Sons. © 2018 WILEY–VCH Verlag GmbH &Co. KGaA, Weinheim. (f) I–V curves of pristine Cs_2_AgBiBr_6_ and PEA-Cs_2_AgBiBr_6_ SC devices. The inset illustrates the schematic of the device’s working mechanism. (g) Comparison of X-ray sensitivities of pristine Cs_2_AgBiBr_6_ and PEA-Cs_2_AgBiBr_6_ SC detector under different applied biases. Reproduced from Ref. 45 © 2019 WILEY–VCH Verlag GmbH & Co. KGaA, Weinheim. (h) Comparison of the X-ray sensitivity of Cs_2_AgBiBr_6_ SCs synthesized by the natural and controlled cooling method under different applied electric fields. Reproduced from Ref.^46^© 2019 WILEY–VCH Verlag GmbH & Co. KGaA, Weinheim. (i) Schematic illustration of the Cs_2_AgBiBr_6_ double perovskite crystal structure (left) and site-specific substitutions (Cs-site, cyan, and Bi-site magenta) (right). (j) Sensitivity for the undoped (middle, labeled “D”) and doped (Cs-site cyan and Bi-site magenta shade) detectors. Reproduced from Ref.^47^ © 2024, American Chemical Society.

Lead-free elpasolites (also known chemically as double perovskites), with the general formula A_2_M^I^M^III^X_6_ (A = monovalent cation, M^I^ = monovalent cation, M^III^ = Bi^3+^, X = halide anion), have gained attention as alternatives to LHPs that maintain the perovskite crystal structure, but without toxic elements that are restricted for use in electronics. Two Pb^2+^ cations are substituted by a combination of one monovalent cation (M^I^) and one trivalent cation (M^III^), thus maintaining the same overall charge as two Pb^2+^ cations would have (presented in Figs. 1, 2, 3). Among bismuth-based double perovskites, the Bi^3+^-Ag^+^ system is one of the most studied set of materials,^11^ particularly Cs_2_AgBiBr_6_. This material has a cubic structure (space group $Fm\overline{3 }m$) with corner sharing metal-halide octahedra, and lattice parameter *a* = 11.27 Å at room temperature. There are several appealing features that are conducive toward high-performance in X-ray detectors. Firstly, Cs_2_AgBiBr_6_ has a *Z*_eff_ value of 53.1, which exceeds those of MAPbBr_3_ (*Z*_eff_ = 45.1) and α-Se (*Z*_eff_ = 34), thus allowing higher X-ray attenuation coefficients (Fig. 4a). Secondly, single crystals of this material have high resistivity (10⁹–10^11^ Ω cm), which exceeds the resistivity of methylammonium lead halide single crystals (10⁷–10⁸ Ω cm). This results in reduced dark and noise current in devices. Furthermore, Cs_2_AgBiBr_6_ has lower field-driven ion migration, which is important for maintaining stable performance with the application of an electric field.^28,29^ Other bismuth-based elpasolites that have been investigated include two-dimensional (2D) (BA)_2_CsAgBiBr_7_ (BA = *n*-butylammonium),^30^ (DFPIP)_4_AgBiI_8_ (DFPIP = 4,4-difluoropiperidinium),^31^ (CPA)_4_AgBiBr_8_ (CPA = chloropropylammonium),^32^ and (PA)_4_AgBiBr_8_ (PA = propylammonium).^33^ These materials are advantageous because of improved stability compared to their inorganic counterparts due to the hydrophobic nature of the long-chain organic A-site cations.

**Table 1:** Key properties of representative bismuth-based materials for X-ray detection.

Materials	Z_eff_	Mass Density (g cm^–3^)	Linear Attenuation efficiency^*a*^	Bandgap (eV)	Reference
Cs_2_AgBiBr_6_	60.0	4.65	99% (1.18 mm, 50 keV)	2.10–2.27	28, 29
BA_2_CsAgBiBr_7_	–	–	99% (20 nm, 70 keV)	2.38	30
(DMEDA)BiI_5_	–	3.83	93.2% (690 μm, 50 keV)	1.86	27
(4,4-DPP)_4_AgBiI_8_	–	2.85	99% (1 mm, 40 keV)	2.03	31
AgBi_2_I_7_	–	–	~ 97% (100 keV, 0.5 mm)	1.73	35
Cs_3_Bi_2_I_9_	–	5.02	94.7% (0.5 mm, 40 keV)	1.94–2.0	36
Rb_3_Bi_2_I_9_	61.6	4.67	90% (30 keV, 0.4 mm)	1.89	37
(NH_4_)_3_Bi_2_I_9_	30.9	4.30	99% (50 keV, 0.99 mm)	2.05	38
(H_2_MDAP)BiI_5_	–	4.36	99.8% (40 keV, 0.4 mm)	1.83	39
MA_3_Bi_2_I_9_	–	3.8–4.1	90% (40 keV, 0.3 mm)	1.98	40
FA_3_Bi_2_I_9_	–	–	99.8% (40 keV, 0.9 mm)	2.08	41
BiI_3_	83.5	5.78	99.8% (50 keV, 1 mm)	1.7–2.2	70
Bi_2_O_3_	79.3	8.9	~ 63% (50 μm, 70 kV, 124 GW)	2.83	43
BiOI	73.6	7.97	90%, (30 keV, 134 μm)	1.93	20

^*a*^The X-ray attenuation efficiency is the fraction of the incident X-ray intensity attenuated within a specified thickness of material

Meanwhile, there are several properties that limit the performance of Cs_2_AgBiBr_6_. In particular, electron–phonon coupling plays an important role. The coupling between charge-carriers and longitudinal optical (LO) phonons (known as Fröhlich coupling) reduces mobilities, while pronounced charge-carrier coupling with acoustic phonons results in small polaron formation. By forming small polarons, charge-carrier mobilities are substantially reduced, limiting CCEs.^48–50^ This carrier localization process arises due to the high acoustic deformation potentials in Cs_2_AgBiBr_6_, and is intrinsic to the material itself. An important question is whether the chemistry of these materials could be changed to lower this acoustic deformation potential.^49^ Various strategies, including doping/alloying, external energy treatments such as thermal annealing, laser/photonic (UV/X-ray/IR exposure) irradiation, plasma and pressure treatments, the use of  heterojunction structures, and bond length compression (using chemical/mechanical pressure) have been attempted to reduce the strength of electron–phonon coupling to increase detector performance.^28,29,51^ For example, Steele and Roeffaers found that annealing Cs₂AgBiBr₆ at 160°C for 1 h led to a reduction of over 10% in the strength of charge-carrier coupling with longitudinal optical phonons (ϒₗₒ), decreasing from 226 to 201 meV.^52^ Similarly, applying pressure (up to 31 GPa) at room temperature in Cs₂AgBiCl₆ resulted in a blue shift of the broad PL emission along with a red shift of the absorption edge. This behavior was attributed to the decreased lattice relaxation energy caused by lattice compression. This significantly reduced Fröhlich coupling, as indicated by the Huang-Rhys parameter, *S*, which is estimated from Stokes shift energy and the LO phonon mode energy $\left( {E_{Stokes} = 2S\hbar \omega_{LO} } \right)$. By increasing the pressure from atmospheric to 4.5 GPa, the *S* value reduced from 18.1 to 8.5. This lattice compression effectively suppressed ionic activity under high pressure, all while preserving the highly symmetric cubic structure.^53^ There has been less work on understanding how the coupling to acoustic phonons could be reduced, but a recent investigation into CuSbSe_2_ suggests that having regular free volume in the structure (by having a layered material), enables reduced acoustic deformation potentials, enabling band-like transport.^54^

### Bismuth-based perovskite derivatives

Vacancy-ordered triple perovskites (VTPs) have also been widely explored, and have the general formula A_3_Bi_2_X_9_, where A refers to the monovalent cation and X is a halogen. These are described as VTPs because the structure can be thought of as derived from a perovskite, where there is a vacancy in the cation site for every three formula units. VTPs typically adopt either a 0D (isolated dimers of Bi_2_X_9_^3−^ surrounded by the A-site cation) or 2D structure (which has corner sharing BiX_6_^3−^ octahedra). As with halide elpasolites, VTPs have been synthesized as SCs, 2D flakes ((NH_4_)_3_Bi_2_I_9_, Rb_3_Bi_2_I_9_), nanocrystals (NCs) (Cs_3_Bi_2_Cl_9_), and quantum dots (MA_3_Bi_2_I_9_), and these have been developed into X-ray detectors.^55–57^

2D-VTPs are obtained by removing every third layer of Bi^3+^ along the (111) direction in the perovskite structure to maintain charge balance (presented in Fig. 1). This layered structure leads to anisotropic electronic properties and X-ray detector performance. Similarly, like 2D VTPs, 0D-A_3_Bi_2_X_9_ VTPs tend to have high effective masses due to the low electronic dimensionality. While this reduces the μT product, it can be beneficial for X-ray detectors by enabling lower dark currents and noise. These 2D/0D VTPs have resistivities ~ 10^12^ Ω cm, which is 2–3 orders of magnitude larger than 3D LHPs, and are comparable to the resistivities of commercial inorganic semiconductors (*e.g*., CZT: 10^9^–10^11^ Ω cm, CdTe: 10^9^ Ω cm), and close to α-Se (10^14^–10^15^ Ω cm).^58^

Furthermore, Bi-based VTPs have high attenuation coefficients for ionizing radiation.^7,59^ For example, the calculated X-ray attenuation coefficient of (NH_4_)_3_Bi_2_I_9_ is comparable to some well-known semiconductors Cs_2_AgBiBr_6_, MAPbBr_3_, CdTe, α-Se and Si.^38^ It is estimated that (NH_4_)_3_Bi_2_I_9_ with 0.99 mm thickness can attenuate 99% of 50 keV X-ray photons (*i.e.*, 99% attenuation efficiency), while MAPbBr_3_ needs 2.28 mm to reach the same attenuation efficiency. Similarly, 2D layered Rb_3_Bi_2_I_9_ more strongly attenuates ionizing radiation than CsPbBr_3_ and Si.^37^ Other VTP variants, including 0D Cs_3_Bi_2_I_9_,^36^ and MA_2_Bi_2_I_9_^40^ also exhibit strong X-ray attenuation. Cs_3_Bi_2_I_9_ SCs with 0.5 mm thickness is able to attenuate 94.7% of the incident X-rays, compared to MAPbI_3_ (87.8%), Cs_2_AgBiBr_6_ (65.9%), MAPbBr_3_ (65.9%) and MAPbCl_3_ (54.2%).^55,60,61^

The low electronic dimensionality of these VTPs results in spatial confinement of charge carriers, enhancing the Coulombic attraction between them, such that the exciton binding energy is high (~ 100 meV), often surpassing the thermal energy (~ 25 meV) at room temperature. This reduces the *μτ* product, as does ion migration. To address these limitations, various strategies, such as surface passivation, blending with higher-dimensional perovskites, and integrating appropriate charge transport layers, should be explored to improve charge-carrier extraction and enhance detectivity.^62^ For example, Zhang et al. employed an epitaxial growth strategy to develop 2D/3D heterocrystals, (BA)₂CsAgBiBr₇/Cs₂AgBiBr₆, for X-ray detection.^63^ The introduction of 2D VTPs induced steric hindrance and increased the activation energy barrier (0.19 eV in the dark) for ion migration.

### Other bismuth-based materials

Apart from structural perovskite derivatives, other classes of Bi-based compounds are appealing because of the similarities in the composition of orbitals at band-edges to LHPs, which is conducive towards achieving defect tolerance.^64,65^ Prominent examples include bismuth oxyiodide (BiOI), bismuth sulfoiodide (BiSI), bismuth iodide (BiI_3_), bismuth chalcogenides (Bi_2_X_3_; X = O, S, Se, and Te), and AgBi_2_I_7_.^35,66^ BiI_3_ and BiOI have a layered structure belonging to the space group *R* $\overline{3 }$ and *P*4/*nmm* (*a* = *b* = 3.99 Å and *c* = 9.21 Å) at room temperature.^42^ BiI_3_ adopts highly polar covalent Bi-I bonds in the layer and weak van der Waals bonding between layers.^42^ BiOI has the stochiometric I-Bi-O-Bi-I layers stacked along the *c*-axis (presented in Fig. 1). Similarly, bismuth chalcogenides have the same rhombohedral structure (*R* $\overline{3 }$ *m*) with a quintuple 2D layer.

In 1999, Dmitriyev et al. ^67^ achieved good resistivity along the *c*-axis of BiI_3_ SCs (ρ ~ 10^8^–10^9^ Ω cm), along with decent *μτ* products of electrons (>10^-5^ cm^2^ V^-1^) and holes (~ 10^-7^ cm^2^ V^-1^). A breakthrough came in 2002, when Matsumoto et al. first reported α-particle detection using an 82 μm-thick BiI_3_ detector with a clear 5.48 MeV peak and an energy resolution of 2.2 MeV FWHM.^68,69^ Since then, there has been much ongoing research into BiI_3_ semiconductors for X-ray detection.^42,68,70^

Similarly, a high effective atomic number *(Z*_eff_ =73.2) coupled with a high mass density of 7.97 g cm^-3^ and high linear attenuation coefficient (10^2^ cm^-1^ at 50 keV) for BiOI makes it a prominent contender for X-ray detection. Only 2% of the incident X-rays generated from a source with 40 kV voltage were transmitted through a 0.4 mm thick stack of BiOI single crystals, while 78% were transmitted through Si of the same thickness.^20^ The photo-excited charge carriers in BiOI couple to intralayer breathing phonon modes, forming large polarons. Unusually for Bi-halide semiconductors, carrier localization is avoided in this material, and free carriers occur at room temperature due to a low exciton binding energy.^20^ At the same time, electron-phonon coupling results in an unavoidable non-radiative loss channel and low carrier mobility at room temperature, which limits the PL lifetime to 2 ns at room temperature, thus limiting diffusion lengths. However, high mobility-lifetime products of 10^-3^ cm^2^ V^-1^ (out-of-plane) and 10^-2^ cm^2^ V^-1^ (in-plane) are still achieved. For example, applying a bias of only 1.8 V across BiOI in the out-of-plane direction (where the mobility-lifetime product is 1.1 × 10^-3^ cm^2^ V^-1^) results in a drift length of 1 mm, exceeding the drift distance required (0.18 mm). We attribute this to the application of an electric field decoupling charge-carriers from the renormalization of the lattice, such that the non-radiative loss channel arising from electron-phonon coupling is avoided, and the drift lifetime then exceeds the diffusion lifetime.

An overall summary of the key properties of these bismuth-based materials discussed here for X-ray detection is shown in Table 1.

### Overall comparison

Having discussed the details of Bi-based compounds explored thus far for X-ray detection, we can draw an overall comparison with established commercial materials and lead halide perovskites (Figs. 2 and 3). As shown in Fig. 2, Bi-based compounds exhibit equivalent or higher attenuation coefficients than Si, α-Se, CdTe, and MAPbI_3_ perovskite. This is due to the high effective atomic numbers of these Bi-based compounds (Table 1), meaning that thinner active layers are required, such that the distance for charge-carrier transport can be shorter.

Beyond a shorter charge-carrier transport distance, achieving high charge collection efficiency also requires a high μτ product. As shown in Fig. 3b, structurally 2D or 3D Bi-based materials exhibit μτ products (10^-4^–10^-2^ cm^2^ V^-1^) that are higher than commercial Si, α-Se (~10^-7^ cm^2^ V^-1^), and comparable to CZT (~10^-3^ cm^2^ V^-1^), and III-V semiconductors, which are expensive to manufacture.^71^

Furthermore, it is important to have high resistivity to effectively suppress noise and dark current, which, together with a high μτ product and attenuation coefficient, increase the signal-to-noise ratio and sensitivity, along with reducing the detection limit of the device. As shown in Fig. 3a, Bi-based compounds exhibit higher resistivity than Si, but comparable resistivity to commercial materials. Notably, the bulk resistivity of 0D Cs_3_Bi_2_Br_9_ VTP SC is higher (~10^12^ Ω.cm), exceeding that of 3D MAPbI_3_ (10^7^–10^10^ Ω.cm) and other commercial materials including Si (10^4^ Ω cm), GaAs (10^8^ Ω cm), CZT (10^10^ Ω cm) and CdTe (10^9^ Ω cm).

Beyond these highly promising materials properties, many of the Bi-based compounds explored thus far have demonstrated high radiation hardness. For instance, Cs_2_AgBiBr_6_ SC has shown remarkable stability under X-ray irradiation with no significant change in dark current even after exposure to doses up to 9.2 Gy_air_, which is equivalent to 92 000 times the dose required for a chest X-ray.^28^ Even in case of AgBi_2_I_7_, high radiation hardness has been observed for continuous X-ray irradiation dose of 58 Gy_air_ (43 keV mean energy), which equals 580 000 times the dose required for a single chest radiograph. After this large dose, there was only a small change in dark current, sensitivity and SNR of the detector.^35,72^ In contrast, CsPbBr_3_ was reported to degrade after exposure to more than 2 Gy_air_ of radiation, suffering a loss of spectral resolution, and requiring post-annealing to recover.^73^ Similarly, conventional semiconductors, such as Si and CZT exhibit even lower radiation hardness, showing performance degradation after exposure to just a few grays due to defect formation, resulting in increased noise and reduced detection efficiency under high dose rates.^74^ Compared to these conventional materials, bismuth-based semiconductors exhibit superior radiation hardness, withstanding cumulative doses up to the kGy range without electrical or structural degradation. The origin of the high radiation hardness of Bi-based materials is not yet thoroughly investigated, however, it is likely attributed to the high resistivity that effectively supresses leakage current, and possibly also defect tolerance and self healing in these materials, which allows high resistivities and large μτ products to be maintained.^72,74^

## X-ray detectors with bismuth-based materials

### Bismuth-based double perovskites

Cs_2_AgBiBr_6_ was first reported for X-ray detection by Pan et al., ^28^ who grew SCs by slow cooling from a heated precursor solution. After washing and annealing the surfaces of these SCs to remove impurities, the SCs exhibited a high resistivity of 1.6 × 10^11^ Ω cm, with a trap density of 1.74 × 10⁹ cm⁻^3^ (Fig. 4b), as determined from space charge-limited current density (SCLC) measurements.^75^ The corresponding charge-carrier mobility was estimated to be 11.81 cm^2^ V^−1^ s^−1^ using Mott–Gurney law.^76^ The sensitivity of the detector with the structure of Au/ Cs_2_AgBiBr_6_ SC/Au was measured to be 105 µC Gy_air_^−1^ cm^−2^ under an electric field of 25 V mm^−1^ (Fig. 4c). However, the performance of their device was poorer than LHPs SC X-ray detectors, and further controlled materials growth and device optimisation will be needed.^77,78^

Steele and co-workers investigated the X-ray detector performance of Cs_2_AgBiBr_6_ double perovskite SCs at both room temperature and low temperature.^44^ The Au/Cs_2_AgBiBr_6_ SC/Au detector (Fig. 4d) exhibited a marked increase in X-ray sensitivity when cooled to liquid nitrogen temperatures, rising from 316 µC Gy_air_⁻^1^ cm⁻^2^ (room temperature) to 988 µC Gy_air_⁻^1^ cm⁻^2^ (liquid nitrogen temperature) under an applied electric field of 50 V mm^−1^ (Fig. 4e). A linear fit to the sensitivity measurements at different temperatures suggests a coefficient of –3.3 µC Gy_air_⁻^1^ cm⁻^2^ K⁻^1^. This rise in sensitivity with decreasing temperature was attributed to 1) an increase in the mobility-lifetime product, and 2) an increase in resistivity (from 5.5 × 10^11^ Ω cm at room temperature to 3.6 × 10^12^ Ω cm at liquid nitrogen temperature). The charge-carrier mobility increase was attributed to reduced electron–phonon scattering, which was also partially responsible for the increase in lifetime from 700 ns (room temperature) to > 1500 ns (liquid nitrogen temperature). Furthermore, reductions in non-radiative recombination at lower temperatures were also considered to contribute to improved lifetimes. The rise in resistivity with a reduction in temperature was attributed simply to a reduction in thermally generated charge-carriers, in addition to reduced non-radiative recombination.^44^

Typically, structural distortion in Cs_2_AgBiBr_6_ SCs can occur due to the disordered arrangement of Ag⁺ and Bi^3^⁺, which arises due to their similar ionic radii, and can negatively impact photoelectric performance. In the fully ordered structure of Cs_2_AgBiBr_6,_ each [AgX_6_]^5−^ octahedron is surrounded by six [BiX_6_]^3−^ octahedra. However, in the case of partial or complete disorder, one or more of these six [BiX_6_]^3−^ octahedra may be replaced by [AgX_6_]^5−^octahedra. Yuan et al. incorporated phenethylammonium bromide (PEABr) into the Cs_2_AgBiBr_6_ perovskite precursor to synthesize PEA-Cs_2_AgBiBr_6_ SCs for X-ray detection.^45^ It was shown that PEABr can effectively suppress the order–disorder phase transition in Cs_2_AgBiBr_6_ SCs, thereby enhancing the X-ray sensitivity of the device. They quantitatively assessed the degree of ordering in Cs_2_AgBiBr_6_ by evaluating the diffraction intensity ratio between the (111) and (022) X-ray diffraction peaks (*I*_111_/*I*_022_). The ordering parameter (*H*) was determined by comparing the ratio of the observed superlattice reflection (111) to the base lattice reflection (022) in the SC with the calculated intensity ratio for a perfectly ordered structure.^79^

5 $$H^{2} = \frac{{\left( {\frac{{I_{111} }}{{I_{022} }}} \right)_{observed} }}{{\left( {\frac{{I_{111} }}{{I_{022} }}} \right)_{calculated} }}$$

As the PEA concentration increases, the *I*_111_/*I*_022_ ratio becomes larger, indicating an enhanced ordering of Bi^3^⁺ and Ag⁺. This suggests that the PEABr precursor effectively promotes cation ordering. The *µτ* value of PEA-Cs_2_AgBiBr_6_ SCs (1.94 × 10^–3^ cm^2^ V^−1^ s^−1^) exceeded that of pristine Cs_2_AgBiBr_6_ SCs (9.14 × 10^−4^ cm^2^ V^−1^ s^−1^) (Fig. 4f). The sensitivity of the detector was also improved up to 288.8 µCGy_air_⁻^1^ cm⁻^2^ at an electric field of 22.7 V mm^−1^ (Fig. 4g).

Chemical treatment applied after growth can further improve crystal quality and enhance detector performance. BiBr_3_ is a crucial precursor in the synthesis of Cs_2_AgBiBr_6_ SCs, and its residue on the double perovskite SC surface contributes to the formation of surface conduction channels.^77^ Zhang and colleagues investigated various post-growth treatment processes and found that rinsing Cs_2_AgBiBr_6_ SCs with isopropanol and applying thermal annealing effectively mitigated field-driven ion migration and surface conduction channels, as well as reducing the detector’s noise current.^77^ The X-ray sensitivity of the detector was estimated to be 316.8 µC Gy_air_^−1^ cm^−2^ at an electric field of 6 V mm^−1^. Yin et al. reported a controlled cooling process for the synthesis of Cs_2_AgBiBr_6_ SC for X-ray detection.^46^ In comparison to the conventional natural cooling method, the controlled cooling process resulted in bismuth-based SC with smooth surfaces, higher resistivity, and improved reproducibility. Thus, the detector exhibits an X-ray sensitivity of 1974 µC Gy_air_^−1^ cm^−2^ under an applied electric field of 50 V mm^−1^ which is higher than the sensitivity value of SC grown by the natural cooling method (Fig. 4h). The SC detector also demonstrates a very low LoDD of 45.7 nGy_air_ s^−1^ at 50 V mm^−1^ applied electric field, surpassing previous reports for Cs_2_AgBiBr_6_ SC X-ray detectors. Donato et al. demonstrated that growing the perovskite in a slightly Bi-deficient and Eu-enriched environment significantly boosts X-ray sensitivity from 17 to 120 μC Gy_air_^–1^ cm^–2^. Furthermore, substituting Cs sites with imidazolium enhances the sensitivity even more, reaching over 180 μC Gy_air_^–1^ cm^–2^ due to higher X-ray attenuation.^47^ Figure 4i presents a schematic illustration of the Cs_2_AgBiBr_6_ crystal structure on the left, alongside site-specific substitutions on the right. Cs-site substitutions are ammonium (A), guanidinium (G), triazolium (T), and imidazolium (Im). Figure 4j shows the trend of the X-ray sensitivity of the pristine (D) and doped samples. The overall increase in sensitivity can be attributed to the fact that lanthanide cations have a K-edge energy value of approximately 50 keV, helping to give to higher X-ray attenuation. Thus, many engineering strategies have been demonstrated to successfully improve the performance of Cs_2_AgBiBr_6_. However, the LoDD and sensitivities reported are currently still at least an order of magnitude inferior to LHPs, and the inherent self-trapping present in Cs_2_AgBiBr_6_ could be a key limiting factor, as discussed earlier. It is therefore important to explore alternative Bi-based materials that could overcome these limitations.

### Bismuth-based 2D perovskites

**Figure 5:**
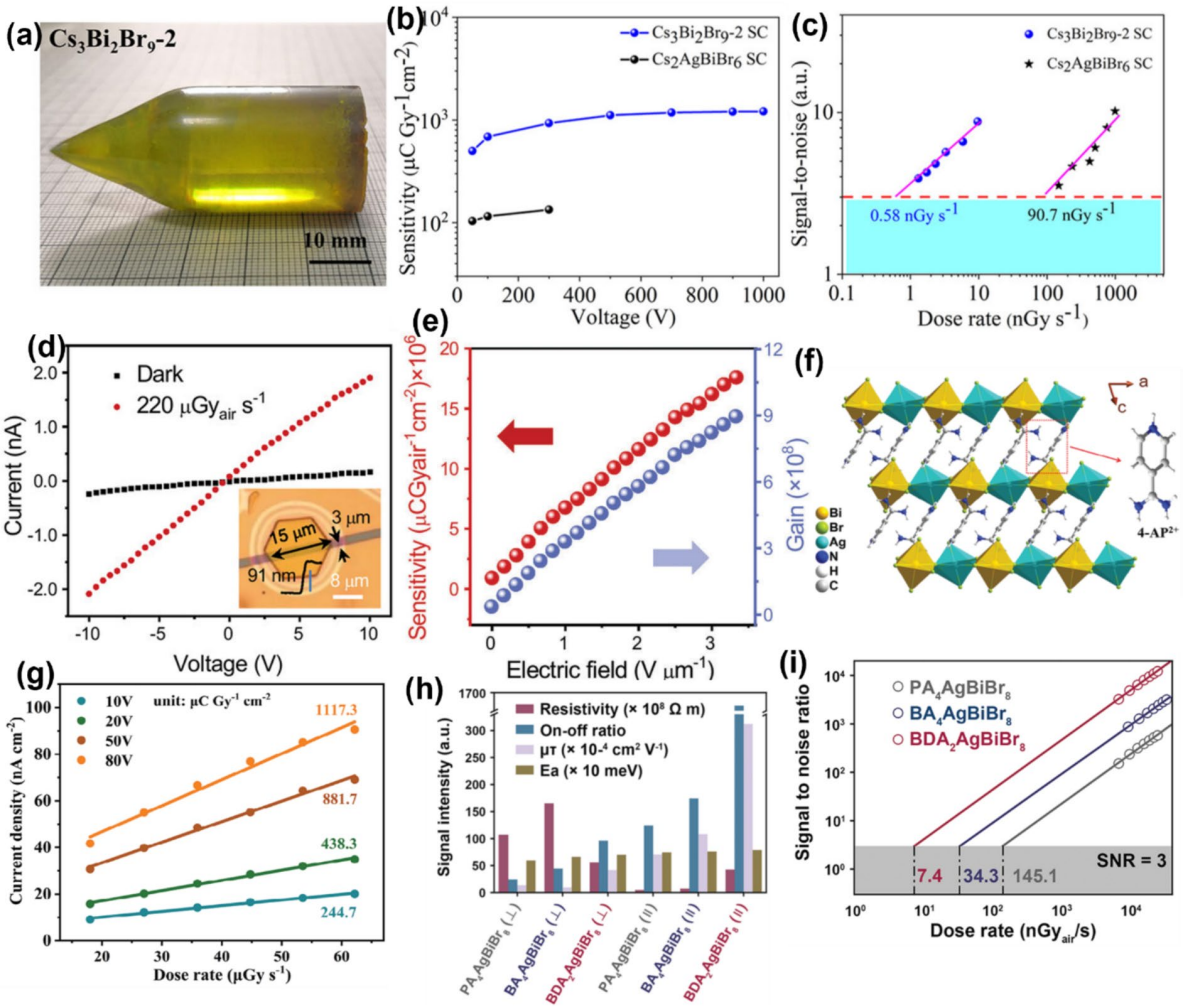
(a) Photograph of as-grown 2D Cs_3_Bi_2_Br_9_ SC grown by the Bridgman method. (b) X-ray sensitivities of Cs_3_Bi_2_Br_9_ and Cs_2_AgBiBr_6_ SC detectors tested under 120 keV hard X-rays. (c) The SNRs of Cs_3_Bi_2_Br_9_ SC and Cs_2_AgBiBr_6_ SC under varying X-ray dose rates. The dashed line indicates an SNR of 3 to measure LoDD. Reproduced from Ref. 83 American Chemical Society © 2022. (d) The dark current and photocurrent of a 2D Cs_3_Bi_2_Br_9_ nanoflake device under X-ray irradiation of 220 µGy s⁻^1^. The inset shows the optical microscopy image of the device. (e) The sensitivity and gain of the detector under different applied electric fields. Reproduced from Ref. 86 John Wiley and Sons © 2023 Wiley‐VCH GmbH. (f) Crystal structure of (4-AP)_2_AgBiBr_8_ 2D perovskite. (g) The variation of photocurrent density of the Ag/(4-AP)_2_AgBiBr_8_ SC/Ag under varying applied voltages and irradiation dose rates. Reproduced from Ref. 87 John Wiley and Sons © 2024 Wiley‐VCH GmbH. (h) Comparison of different detector parameters of (PA)_4_AgBiBr_8_, (BA)_4_AgBiBr_8_, and (BDA)_2_AgBiBr_8_ SC. (i) The X-ray dose rate dependence of SNR of the detectors under an external bias voltage of 200 V. The LoDD is determined from the fitting line corresponding to an SNR of 3. Reproduced from Ref. 88 © John Wiley and Sons 2023 Wiley‐VCH GmbH.

2D Cs_3_Bi_2_Br_9_ VTPs can be an excellent candidate for sensitive X-ray detection due to high X-ray attenuation, and high bulk resistivity with a decent mobility-lifetime product (Table 1). The layered structure of Cs_3_Bi_2_Br_9_ VTP results in anisotropic electronic properties. The limited carrier transport in the out-of-plane direction of the SC helps to reduce noise and lower dark current drift due to ion migration in vertically structured devices, ultimately contributing to a lower LoDD. However, the vertical device can exhibit lower X-ray sensitivity compared to the planar device due to its reduced mobility. Saqr et al. reported solution-grown Cs_3_Bi_2_Br_9_ SCs for direct X-ray detection.^80^ Ag/Cs_3_Bi_2_Br_9_ SC/Ag vertical devices exhibited a resistivity of 1.79 × 10^11^ Ω cm and *μτ* product of 5.12 × 10^−4^ cm^2^ V^−1^. Compared to the low-temperature solution-growth technique, the high-temperature melt growth method offers significant advantages in producing high-quality, large-area SCs.^81,82^ This approach enables the formation of crystals with superior structural integrity and substantially reduced defect density. Xiang and co-workers reported high-quality 2D Cs_3_Bi_2_Br_9_ VTP SCs grown from a melt via the Bridgman method shown in Fig. 5a.^83,84^ The bismuth-based perovskite SC exhibited a high resistivity of 1.41 × 10^12^ Ω cm and mobility-lifetime product of 8.32 × 10^–4^ cm^2^ V^−1^. The Au/Cs_3_Bi_2_Br_9_ SC/Au device demonstrates impressive sensitivity, achieving 1705 µC Gy_air_^−1^ cm^−2^ under an applied electric field of 1000 V mm^−1^ (Fig. 5b), along with an exceptionally low detection limit of 0.58 nGy_air_ s^−1^ for detecting 120 keV hard X-rays (Fig. 5c). The Cs_3_Bi_2_Br_9_ detector also shows remarkable operational stability, featuring a minimal dark current drift of 2.8 × 10^–10^ nA cm^−1^ s^−1^ V^−1^ and long-term stability in air under a high electric field of 1000 V mm^−1^, attributed to the high activation energy barrier to ion migration, which arises as a result of its 2D structure. Thus, the device performance of bismuth-based 2D perovskite SCs is on par with that of conventional 3D LHP SCs.^85^

Zhi synthesized 2D Cs_3_Bi_2_Br_9_ nanoflakes using inversion temperature crystallization (ITC) for high-performance X-ray detection.^86^ Without the addition of AgBr in the precursor solution, the nanoflakes show a rectangular morphology with CsBiO_3_ impurities. After adding AgBr to the precursor, the Br^−^ vacancies in the lattice of Cs_3_Bi_2_Br_9_ were passivated. Br atoms at the vertices of [BiBr_6_]⁻ octahedra in Cs_3_Bi_2_Br_9_ can easily migrate, creating Br^−^ vacancies. These vacancies trap electrons and destabilize the structure, making it prone to oxidation in HBr solution, leading to the formation of CsBiO_3_ and Cs_3_Bi_2_Br_9_ hybrids. Adding AgBr to the precursor solution reduces Br⁻ vacancies by filling trap states and inhibiting Br migration, thus enhancing structural stability. The synthesized highly crystalline 2D Cs_3_Bi_2_Br_9_ nanoflakes exhibit a direct bandgap, with a mobility-lifetime product reaching 9.8 × 10⁻^4^ cm^2^ V⁻^1^. Notably, devices constructed from these 2D nanoflakes demonstrate a high sensitivity of 1.9 × 10^5^ μC Gy_air_^−1^ cm^−2^, attributed to photoconductive gain. Figure 5d shows the comparison of the dark current and X-ray photocurrent of 2D Cs_3_Bi_2_Br_9_ nanoflake detectors under X-ray irradiation with a dose rate of 220 µGy_air_ s^−1^, and the optical micrograph of the device is inset. The sensitivity of the detector increases up to 1.9 × 10^6^ µC Gy_air_^−1^ cm^−2^ as the electric field increases from 0 to 3.3 V µm^−1^ as shown in Fig. 5e. The gain of the detector is approximately 8.9 × 10^8^, confirming that the photoconductive gain mechanism contributes to the exceptional sensitivity of devices made from 2D Cs_3_Bi_2_Br_9_ nanoflakes (Fig. 5e). While photoconductive gain can enhance sensitivity, it also increases the noise current, which in turn restricts the LoDD. Li et al. demonstrated Cs_3_Bi_2_Br_9_ thick films grown by CVD.^89^ The vertical device structure, with the configuration of Au/ Cs_3_Bi_2_Br_9_ films /SnO_2_/ITO, demonstrates good X-ray sensitivity of 593 μC Gy_air_^−1^ cm^−2^, a low detection limit of 187.7 nGy_air_ s^−1^, and outstanding stability, maintaining performance after 20 days in ambient conditions and during continuous operation for over 2 h.

X-ray detection without an applied bias is highly desirable for developing energy-efficient, portable detectors, with potential applications in biomedical imaging, radiation dose monitoring, and security scanning for remote or inaccessible locations. Wu and co-workers demonstrated a chirality-induced polar photovoltaic effect in a chiral-polar 2D bismuth-based perovskite (R-MPA)_4_AgBiI_8_ (R-MPA = R-*β*-methylphenethylammonium) SCs for self-powered X-ray detection.^90^ The strong spontaneous electric polarization in bismuth-based SC results in a notable polar photovoltage of 0.36 V, which facilitates the separation and transport of X-ray-generated charge-carriers, enabling self-powered detection. As a result, X-ray detectors constructed from high-quality SCs of (R-MPA)_4_AgBiI_8_ demonstrate a high sensitivity of 46.3 μC Gy_air_^–1^ cm^–2^ and a low LoDD of 85 nGy_air_ s^–1^ at zero bias. The performance of this self-powered X-ray detector was comparable to that of other reported lead-based 2D chiral-polar perovskite SCs.^91–93^ This sensitivity can be further enhanced to 949.6 μC Gy_air_^–1^ cm^–2^ when applying a bias of 50 V across the electrodes.

Solution-grown bismuth-based layered 2D hybrid double perovskite SCs have also been reported for X-ray detection.^30,31,94^ Wu et al. reported bismuth-based (4-AP)_2_AgBiBr_8_ (4-AP = 4-amidinopyridine) Dion-Jacobson (DJ) 2D perovskite SC for sensitive X-ray detection (Fig. 5f).^87^ In this DJ structure, the AgBr₆ and BiBr₆ octahedra are alternately arranged and corner sharing, creating a 2D inorganic monolayer. Figure 5g shows the variation of the photocurrent density of the Ag/(4-AP)_2_AgBiBr_8_ SC/Ag detector with X-ray irradiation dose rates under different applied bias voltages. The slope of the linear fit represents the sensitivity of the detector. The SC detector exhibits a high sensitivity value of 1117.3 μC Gy_air_^–1^ cm^–2^ under applied bias of 80 V. Huanyu and co-workers presented the comparison of X-ray detection performance of three different layered hybrid silver bismuth bromine SCs: (BDA)_2_AgBiBr_8_ (BDA = 1,4-diaminobutane), (BA)_4_AgBiBr_8_ (BA = *n*-butylamine), PA_4_AgBiBr_8_ (PA = *n*-propylamine).^88^ Figure 5h presents the comparison of different device parameters of three different SCs in the in-plane (parallel) and out-of-plane (perpendicular) directions. The (BDA)_2_AgBiBr_8_ SC demonstrated a high *μτ* value, bulk resistivity, and an excellent on/off ratio, making it a promising candidate for X-ray detection. In optimized in-plane devices, the detectors based on (BDA)_2_AgBiBr_8_ achieved a sensitivity of 2638 µC Gy_air_⁻^1^ cm⁻^2^ and an exceptionally low detection limit of 7.4 nGy_air_ s⁻^1^ (Fig. 5i). 2D bismuth-based (F-PEA)_3_BiI_6_ [(F-PEA) = 4-fluorophenethylammonium] pressed wafer with an area of 1.33 cm^2^ was reported with sensitivity of 52.6 µC Gy_air_⁻^1^ cm⁻^2^ and LoDD of 30 nGy_air_ s^−1^.^95^ It is important to note that large-area devices are highly desirable for the fabrication of multi-pixel X-ray imagers. Thus, low-cost solution-grown bismuth-based layered perovskites could be promising candidates for X-ray detection.

### Bismuth-based 0D perovskites

**Figure 6:**
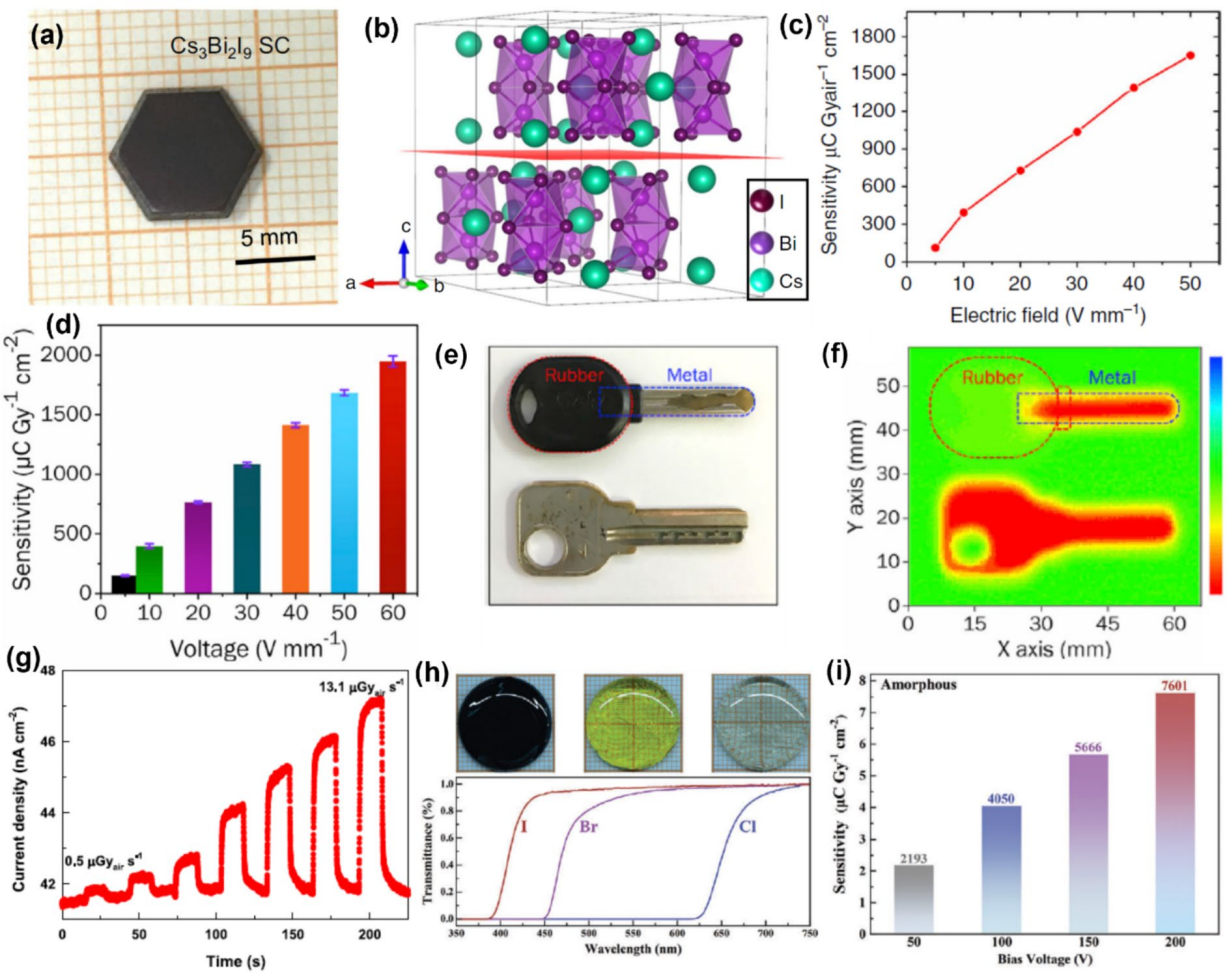
(a) Photographs of as-grown Cs_3_Bi_2_I_9_ 0D SC. (b) Crystal structure of a 2 × 2 × 1 supercell of Cs_3_Bi_2_I_9_. (c) X-ray sensitivity of the detector under different applied electric fields. Reproduced from Ref. 36 Springer Nature Copyright © 2020. (d) X-ray sensitivity of the MA_3_Bi_2_I_9_ SC detector under different applied electric fields. (e) Photograph and (f) corresponding X-ray images obtained from MA_3_Bi_2_I_9_ SC detector. Reproduced from Ref. 96 © 2020 Elsevier Inc. (g) Temporal response of Au/FA_3_Bi_2_I_9_/Au detector to X-rays with different dose rates under a bias of 180 V. Reproduced from Ref. 41 Copyright © 2021, American Chemical Society (h) Photographs and UV–vis transmittance spectra of MTP_3_Bi_2_Cl_9_, MTP_3_Bi_2_I_9_ and MTP_3_Bi_2_I_9_ wafers. (i) X-ray sensitivity of Au/MTP_3_Bi_2_I_9_ amorphous wafer/Au detector under different applied bias. Reproduced from Ref. 97 © 2024 Wiley‐VCH GmbH.

Zhang et al. developed a nucleation-controlled solution method to synthesize large-size high-quality Cs_3_Bi_2_I_9_ perovskite zero-dimensional (0D) SCs as shown in Fig. 6a.^36^ After filtration, the CsI and BiI_3_ precursor solution was placed in a temperature-controlled oven. The temperature was raised to 80°C, such that sub-millimeter Cs_3_Bi_2_I_9_ SCs were precipitated out. To eliminate nucleation seeds, the system was maintained at this temperature for 24 h. Once the solution reached saturation, the excess material recrystallized. The supernatant was carefully transferred to a new container to grow large single crystals. The structure of these VTPs was discussed earlier in Sect. “Bismuth-based perovskite derivatives”. The SC exhibited a high resistivity of 2.79 × 10^10^ Ω cm with a μτ value of 7.97 × 10^–4^ cm^2^ V^−1^, and devices achieved a sensitivity of 1652.3 μCGy_air_^−1^ cm^−2^ at 50 V mm^−1^ applied electric field (Fig. 6c, with an LoDD of 130 nGy_air_ s^−1^. Liu et al. reported inch-sized 0D MA_3_Bi_2_I_9_ (MA = CH_3_NH_3_) SCs grown by solution processing method for sensitive X-ray detection and imaging.^96^ The bismuth-based SC detector demonstrated a high resistivity of 3.74 × 10^10^ Ω cm and a substantial *μτ* product of 2.87 × 10^−3^ cm^2^ V^−1^. The SC X-ray detector exhibited a very high sensitivity of 1947 μC Gy_air_^−1^ cm^−2^ under an electric field of 60 V mm^−1^ (Fig. 6d), a low detection limit of 83 nGy_air_ s^−1^, and a short response time of 23.3 ms. Additionally, the bismuth-based SC is utilized further for demonstration of X-ray imaging as shown in Fig. 6e–f. Zheng reported bismuth-based MA_3_Bi_2_I_9_ SCs with a high sensitivity of 10620 µC Gy_air_^−1^ cm^−2^ and ultra-low LoDD of 0.62 nGy_air_ s^−1^.^14^ The coplanar detector made from MA_3_Bi_2_I_9_ SC demonstrates impressive performance, including a low dark noise current and exceptional X-ray response.^40^ Charge-carrier transport within the MA_3_Bi_2_I_9_ SC differs along the [010] and [001] directions due to scattering effects. In a coplanar device, carriers transfer along the [Bi_2_I_9_]^3−^ intralayer, while in a vertical device, they must travel through the interlayer along the [001] direction. During the growth of the SC, potential barriers such as ion vacancies, traps, and disorder states are typically introduced into the interlayer between the [Bi_2_I_9_]^3−^ monolayers. These act as scattering centers, which reduce carrier mobility. As a result, carrier mobility is expected to be higher in a coplanar structure than in a vertical one. It features a high sensitivity of 872 μC Gy_air_⁻^1^ cm⁻^2^, a rapid response time of 266 μs, and a low detection limit of 31 nGy_air_ s⁻^1^.^40^ Additionally, the detector offers a spatial resolution of 4.22 lp mm⁻^1^ and long-term stability, with a small area single pixel device. Li and co-workers reported FA_3_Bi_2_I_9_ (FA = CH(NH_2_)_2_) SCs grown by the nucleation-controlled secondary solution constant temperature evaporation (SSCE) method.^41^ The temporal X-ray response of Au/FA_3_Bi_2_I_9_/Au detector to X-rays with dose rates from 0.5 to 13.1 μGys^−1^ under a bias of 180 V is shown in Fig. 6g. The SC crystal detector exhibited a sensitivity of 598.1 μC Gy_air_^−1^ cm^−2^ and an LoDD of 0.2 μGy_air_ s^−1^, which are improved over commercial α-Se detectors.

It is important to note that large-area films and wafers are highly desired for the production of flat-panel X-ray detectors, which are essential for X-ray imaging applications. Polycrystalline bismuth-based 0D perovskite thick films and pellets have also been reported for X-ray detection.^61,98^ Xu and co-workers reported a high-quality large-area amorphous MTP_3_Bi_2_X_9_ (methyltriphenylphosphonium = MTP and X = Cl, Br, or I) wafers grown by the melt-quenching method.^97^ Figure 6h depicts the photographs and ultraviolet–visible (UV–vis) transmission spectra of the MTP_3_Bi_2_Cl_9_, MTP_3_Bi_2_I_9,_ and MTP_3_Bi_2_I_9_ wafers. MTP_3_Bi_2_I_9_ amorphous wafer exhibited high X-ray sensitivity of 7601 μC Gy_air_^−1^ cm^−2^ under 200 V of applied bias (Fig. 6i). However, further research is needed to fabricate and optimize multipixel, large-area X-ray imagers using this bismuth-based material for potential commercial applications.

## Other bismuth-based materials for X-ray detection

**Figure 7:**
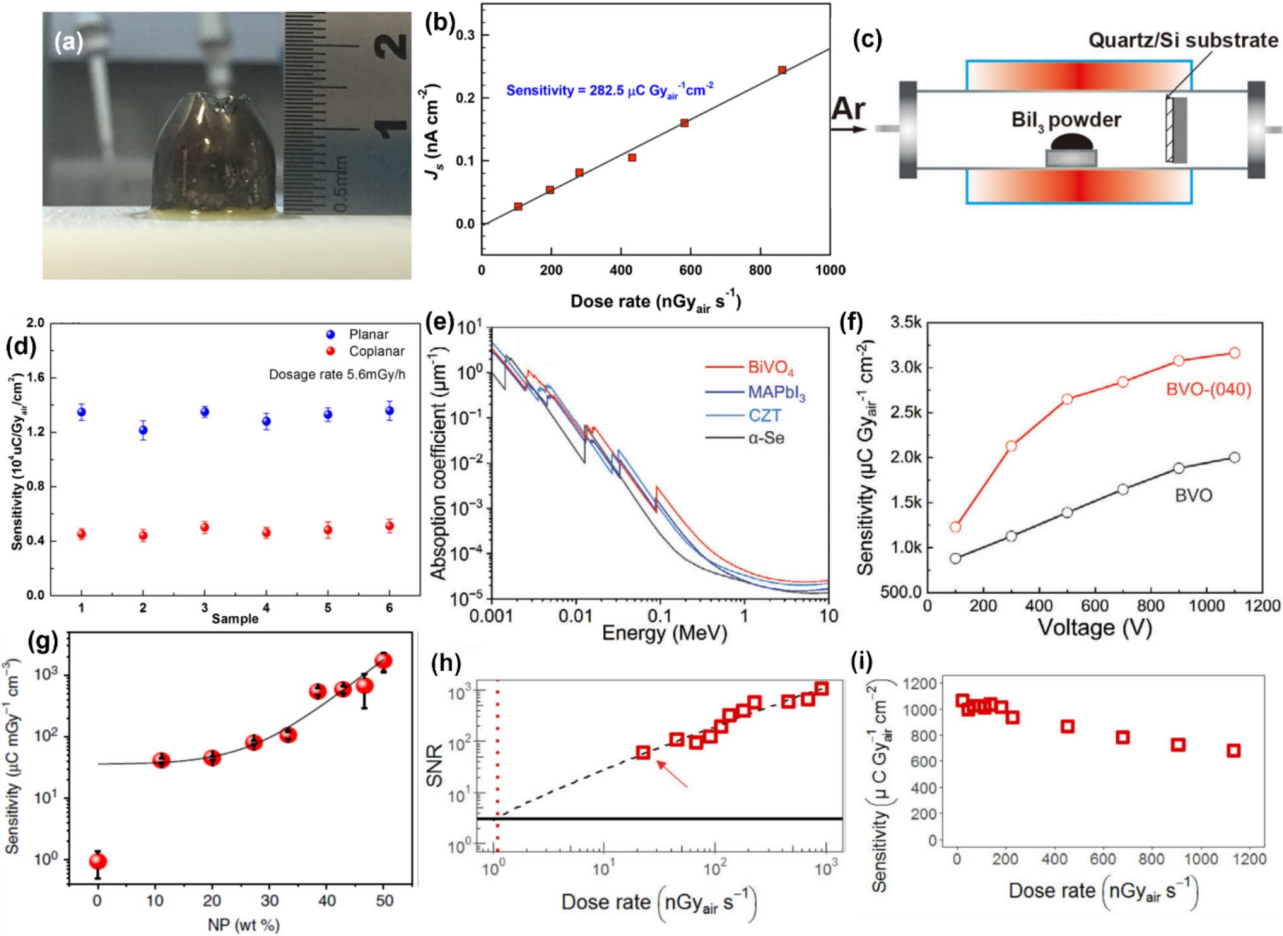
(a) Photograph of the AgBi_2_I_7_ crystals grown by the vertical Bridgman technique. (b) Current density (*J*_s_) of the AgBi_2_I_7_ detector as a function of irradiation dose rate. The slope of the linear fit corresponds to the sensitivity of the device. Reproduced from Ref. 35 Copyright © 2020, American Chemical Society. (c) Schematic of the fabrication process of BiI_3_ SC via the physical vapor transport method. (d) Sensitivity of multiple BiI_3_ detectors. Reproduced from Ref. 99 Copyright © 2023 Wiley–VCH GmbH. (e) Comparison of the attenuation coefficient of BiVO_4_ as a function of photon energy with MAPbI_3_, CZT, and α-Se. (f) X-ray sensitivity of the BiVO_4_ detector as a function of applied bias. Reproduced from Ref. 101 Copyright © 2018 Springer Nature. (g) Variation of X-ray sensitivity of ITO/PEDOT:PSS/P_3_HT:PC_70_BM:Bi_2_O_3_/Al device with Bi_2_O_3_ nanoparticle loading. Reproduced from Ref. 102 Copyright © 2018 Springer Nature. (h) SNR of Au/BiOI/Au SC detector as a function of X-ray dose rate, and the dashed red line shows the corresponding dose rate (1.1 nGy_air_ s^−1^) at which the SNR value is 3. (i) X-ray sensitivity of the perpendicular BiOI SC detector as a function of dose rates. Reproduced from Ref. 20 Copyright © 2023 Springer Nature.

In addition to bismuth-based perovskite materials, high-performance X-ray detectors have also been reported using Bi-based compounds without the perovskite structure, including AgBi₂I₇ SCs, BiI₃, Bi₂O₃, and layered BiOI SCs. Tie et al. reported AgBi_2_I_7_ SCs (Fig. 7a) grown by a vertical Bridgman technique.^35^ The *μτ* values of the SC detector were 3.4 × 10^–3^ and 1.2 × 10^–3^ cm^2^ V^–1^ for electrons and holes, respectively. The AgBi_2_I_7_ SC exhibited very high mobility values of 492.1–859.3 cm^2^ V^–1^ s^–1^ and 296.2–702.5 cm^2^ V^–1^ s^–1^ for electrons and holes, respectively. The X-ray sensitivity of the Au/AgBi_2_I_7_ SC/Au SC detector was obtained to be 282.5 μC Gy_air_^–1^ cm^–2^ (Fig. 7b) while the detector demonstrated a LoDD of 72 nGy_air_ s^–1^. Sun and co-workers demonstrated free-standing BiI_3_ SC flakes grown by the physical vapor transport method as shown in Fig. 7c.^99^ The SC detector exhibited very high sensitivity of 1.22–1.36 × 10^4^ µCGy_air_^−1^ cm^−2^ along [001] direction (Fig. 7d). These enhancements in the measured sensitivity can also result from photoconductive gain. Au/BiI_3_ SC/Au X-ray detector was reported with a high signal-to-noise ratio of 896.4 and a sensitivity up to 0.526 × 10^4^ μC Gy_air_^−1^ cm^−2^ along the c*-*axis direction under an electric field of 0.02 V μm^−1^ and X-ray dose rate of 489.78 μGy h.^−1^.^100^

Fan and co-workers recently reported bismuth vanadate (BiVO_4_) sintered pellets for 110 kVp hard X-ray detection.^101^ The comparison of the X-ray attenuation coefficient with the photon energy of BiVO_4_ with other detector materials is presented in Fig. 7e. BiVO_4_ offers excellent X-ray attenuation, particularly for photons with energies greater than 90 keV. At a thickness of 1 mm, BiVO₄ achieves an impressive attenuation efficiency of 97.1%, significantly outperforming other materials, which remain below 80%. Ultra-stable BiVO₄ metal oxide X-ray detectors exhibit a high sensitivity of 3164 μC Gy_air_⁻^1^ cm⁻^2^ and a low detection limit of 20.76 nGy_air_ s⁻^1^ under 110 kVp hard X-rays, setting a new benchmark for X-ray detectors based on polycrystalline Bi-halides and metal oxides. BiVO_4_ pellet X-ray detector was reported with a large resistivity of 1.3 × 10^12^ Ω cm, negligible current drift of 6.18 × 10^−8^ nA cm^−1^ s^−1^ V^−1^, a high *µτ* value of 1.75 × 10^−4^ cm^2^ V^−1^, an X-ray sensitivity of 241.3 µC Gy_air_^−1^ cm^−2^ and a detection limit of 62 nGy_air_ s^−1^ under 40 kVp X-ray illumination.^103^ Ceramic BiVO_4_ wafers exhibit lower charge-carrier mobility and mobility-lifetime products than bismuth-based perovskite single crystals, mainly due to their polycrystalline nature. This limitation in electronic properties can impact the overall efficiency and performance of detectors utilizing BiVO₄ ceramics. Additionally, the fabrication process for BiVO₄ ceramic wafers involves multiple complex steps. One of the critical stages is energy-intensive high-temperature sintering, conducted within a temperature range of 650 to 800°C.

Furthermore, Praveenkumar et al. synthesized phase-pure Bi_5_O_7_I NCs, and X-ray detectors based on this achieved a sensitivity of 1.92 ×10^-2^ μC Gy_air_^-1^cm^-2^.^104,105^ Due to their low cost and compatibility with solution processing, organic semiconductors based on conjugated polymers and small molecules hold promise for use in X-ray detectors, particularly flexible, wearable detectors. However, their performance is limited by inherently poor X-ray attenuation. To address this limitation, inorganic nanomaterials with high atomic numbers can be incorporated to enhance the sensitivity of organic semiconductor devices for ionizing radiation detection. Jayawardena et al. showed a direct X-ray detector on Bi_2_O_3_ nanoparticles dispersed in poly(3-hexylthiophene-2,5-diyl) (P3HT) and [6,6]-phenyl C_71_ butyric acid methyl ester (PC_70_BM) with sensitivity 160 μC mGy_air_^−1^ cm^−3^ exhibits almost 100% attenuation.^43^ Thirimanne et al. incorporated bismuth oxide (Bi_2_O_3_) nanoparticles with high atomic number into an organic bulk heterojunction for X-ray detection.^43,102^ Figure 7g depicts the variation of X-ray sensitivity of ITO/PEDOT:PSS/P_3_HT:PC_70_BM:Bi_2_O_3_/Al device with Bi_2_O_3_ nanoparticle loading. These hybrid detectors demonstrated X-ray sensitivities of 1712 µC mGy_air_^−1^cm^−3^ for soft X-rays and ~30 and 58 µC mGy_air_^−1^ cm^−3^ under 6 and 15 MV hard X-rays.

Recently, Jagt et al. demonstrated layered bismuth oxyiodide (BiOI) SCs for X-ray detection with low LoDD. As discussed in Section “Key properties of bismuth-based materials for X-ray detection”, BiOI is unusual among Bi-halide materials by having band-like transport, enabling high mobility-lifetime products of 1.1× 10^−3^ cm^2^ V^-1^ (out-of-plane) and 6 × 10^−2^ cm^2^ V^−1^ (in-plane).^20^ These SC detectors exhibited ultra-low LoDD of 1.1 nGy_air_ s^−1^ (Fig. 7h) and a high sensitivity of 1.1 × 10^3^ μC Gy_air_^−1^ cm^−2^ under 5 V applied bias in the out-of-plane direction (Fig. 7i). Despite exhibiting band-like charge-carrier transport, BiOI still suffers from non-radiative losses due to strong electron-phonon coupling. Its layered crystal structure limits single-crystal thickness results in anisotropic growth, such that SCs are only a few hundred microns thick (despite having lateral dimensions > 5 mm), which is insufficient for high stopping power with high-energy X-rays. Additionally, the low yield, high temperature processing, and challenges with large crystal growth limit the practical applications of these SCs. As a result, polycrystalline BiOI wafers or thick films offer a more viable alternative. They allow for greater thickness and area coverage, improving X-ray attenuation, and are compatible with scalable fabrication, and should be explored in the future.

The surface of SCs usually contains a large number of dangling bonds, under-coordinated atoms, surface dislocations, and chemical impurities.^106^ There are notable differences in the physical properties between the surface and bulk of SCs. Therefore, surface passivation and heterojunction formation can be effective strategies to improve detection performance. Recently, solution-grown thick BiI/BiI_3_/BiI (Bi_x_I_y_) van der Waals heterostructures were reported with a sensitivity up to 4.3 × 10^4^ μC Gy_air_^−1^ cm^−2^ and a detection limit as low as 34 nGy_air_ s^−1^.^70^ Therefore, bismuth iodide and various oxide materials show significant promise for the development of highly sensitive X-ray detection and imaging technologies. A summary of the X-ray sensitivity, LoDD, and other device parameters for various bismuth-based materials is presented in Table 2.

**Table 2:** Summary of the performance of X-ray detectors made from bismuth-based materials.

Device configuration	μτ product (cm^2^ V^−1^)	Resistivity (Ω cm)	Electric field (V mm^−1^)	LoDD (nGy_air_ s^−1^)	Sensitivity (µC Gy_air_^−1^ cm^−2^)	Reference
Au/Cs_2_AgBiBr_6_ SC/Au	6.3 × 10^−3^	1.6 × 10^11^	25	59.7	105	28
Au/Cs_2_AgBiBr_6_ SC/Au	–	3.6 × 10^12^	50	–	316 at 300 K 988 at 77 K	44
Au/PEA-Cs_2_AgBiBr_6_ SC/Au	1.94 × 10^−3^	–	22.7	–	288.8	45
Au/Cs_2_AgBiBr_6_ SC/Au	–	–	6	–	316	77
Au/Cs_2_AgBiBr_6_ SC/Au	5.95 × 10^−3^	3.31 × 10^10^	50	45.7	1974	46
Au/Imidazolium substituted Cs_2_AgBiBr_6_ SC/Au	–	–	Bias = 10 V	–	180	47
Au/Cs_2_AgBiCl_6_ SC/Au	5.36 × 10^–4^	3.1 × 10^10^	40	< 241	325.8	107
Au/ BiOBr passivated Cs_2_AgBiBr_6_ wafer/Au	5.51 × 10^−3^	1.6 × 10^10^	500	95.3	250	108
Au/Cs_2_AgBiBr_6_ film/Au	–	–	–	145.2	1.8 × 10^4^	109
Au/Cs_2_AgBiBr_6_:PVA film/Au flexible device	–	2.0 × 10^11^	4000	–	40	110
Au/ Cs_3_Bi_2_Br_9_ SC /Au	8.32 × 10^−4^	1.41 × 10^12^	1000	0.58	1705	83
Au/ Cs_3_Bi_2_Br_9_ SC /Au	–	6.8 × 10^11^	100	–	230.4	84
Ag/ Cs_3_Bi_2_Br_9_ SC /Ag	5.12 × 10^−4^	1.79 × 10^11^	–	–	–	80
Au/Cs_3_Bi_2_I_6_Br_3_ SC/Au	–	2.3 × 10^10^	30	–	55.62	81
Au/2D Cs_3_Bi_2_Br_9_ nanoflakes/Au	9.8 × 10^−4^	1.6 × 10^7^	7142	–	1.9 × 10^5^	86
ITO/SnO_2_/Cs_3_Bi_2_Br_9_ films /Ag	–	–	~ 625	187.7	593	89
Au/(BA)_2_CsAgBiBr_7_ SC/Au	1.21 × 10^−3^	–	5	–	4.2	30
Au/(4,4-DPP)_4_AgBiI_8_ SC/Au	–	–	Bias = 50 V	3130	188	31
Ag/(I-BA)_4_AgBiI_8_ SC/Ag	2.28 × 10^−3^	3.04 × 10^10^	4.5	–	5.38	94
Ag/(R-MPA)_4_AgBiI_8_ SC/Ag	2.2 × 10^−5^	1.54 × 10^10^	0	85	46.3	90
Ag/(4-AP)_2_AgBiBr_8_ SC/Ag	4.8 × 10^–4^	1.7 × 10^11^	Bias = 80 V	279	1117.3	87
Carbon/(BDA)_2_AgBiBr_8_/Carbon	3.12 × 10^–2^	4.25 × 10^9^	Bias = 200 V	7.4	2638	88
Ag/(H_2_MDAP)BiI_5_ SC/Ag	–	2.1 × 10^10^	5	–	1.0	39
Au/(F-PEA)_3_BiI_6_ wafer /C60/ BCP/Cr	8.3 × 10^–5^	2 × 10^11^	100	30	52.6	95
Au/Cs_3_Bi_2_I_9_ SC/Au	7.97 × 10^–4^	2.79 × 10^10^	50	130	1652.3	36
Au/ MA_3_Bi_2_I_9_ SC/Au	2.87 × 10^−3^	3.74 × 10^10^	60	83	1947	96
Au/MA_3_Bi_2_I_9_ SC/Au	2.8 × 10^−3^	5.27 × 10^11^	~ 120	0.62	10,620	14
Au/MA_3_Bi_2_I_9_ SC/Au	–	4.7 × 10^10^	2860	31	872	40
ITO/MA_3_Bi_2_I_9_ film/Au	3.89 × 10^–5^	5 × 10^11^	136	140	35	98
Au/ MA_3_Bi_2_I_9_ pellet/Au	4.6 × 10^−5^	2.28 × 10^11^	210	9.3	563	61
Au/FA_3_Bi_2_I_9_ SC/Au	2.4 × 10^−5^	7.8 × 10^10^	555	200	598	41
Au/Rb_3_Bi_2_I_9_ SC/Au	2.51 × 10^−3^	2.3 × 10^9^	300	8.32	159.7	37
Au/ MTP_3_Bi_2_I_9_ wafer/Au	1.88 × 10^−4^	–	400	19.69	7601	97
Ag/ (NH_4_)_3_Bi_2_I_9_ SC/Ag	4.0 × 10^−3^	–	6.5	55	803	38
Ag/ (HIS)BiI_5_ SC/ Ag	2.81 × 10^−4^	2.31 × 10^11^	2.5	36.4	10^3^	111
Au/AgBi_2_I_7_ SC/Ag	1.2 × 10^−3^	1.3 × 10^8^	0.38	72^61^	282.5	35
Au/BiI_3_ SC/Au	–	6.4 × 10^11^	–	–	1.36 × 10^4^	99
Au/BiI_3_ SC/Au	–	3.43 × 10^11^	20	–	5.26 × 10^3^	100
Ag/ PMMA polystyrene-BiI_3_/Ag	–	–	1	5000	189 µC Gy^−1^ cm^−3^	112
Ag/BiVO_4_ pellet /Ag	1.15 × 10^−4^	3.61 × 10^11^	1100	20.76	3164	101
Au/ BiVO_4_ pellet/ Au	1.75 × 10^–4^	1.3 × 10^12^	62.3	62	241.3	103
Al/BCP/P_3_HT:PC_70_BM:Bi_2_O_3_ nps /PEDOT:PSS/ITO	–	–	~ 500	–	1712 µC mGy^−1^ cm^−3^	102
Au/ P3HT:PCBM:Bi_2_O_3_ pellet/Au	1.7 × 10^–6^	–	1200	–	160 μC mGy^−1^ cm^−3^	43
Au/ BiOI SC/Au	(1.1 ± 1.4) × 10^−3^	1.1 × 10^12^	2780	1.1	1100	20
Cu/Bi_x_I_y_/Cu	3.0 × 10^−3^	4.1 × 10^9^	24	34	4.3 × 10^4^	70

## Conclusions and outlook

In conclusion, Bi-based compounds exhibit several appealing properties for X-ray detection that have sparked a resurgence of interest in this area. The composition of heavy elements, and the high mass density of these materials lead to strong attenuation of ionizing radiation. Combined with the high mobility-lifetime products (reaching > 10^–2^ cm^2^ V^−1^ in some cases) and low dark current densities, high sensitivities (> 10^4^ μC Gy_air_^−1^ cm^−2^) and low LoDD < 10 nGy_air_ s^−1^ have been achieved in Bi-based materials used in direct X-ray detectors. The high versatility of these materials is such that the structural dimensionality can be tuned from 3D (*e.g.*, Cs_2_AgBiBr_6_) to 0D (*e.g.*, (MA)_3_Bi_2_I_9_). Owing to higher effective masses, low-dimensional materials, especially VTPs, benefit from reduced dark currents that are conducive toward achieving lower LoDDs. Bi-based compounds, in addition to having low toxicity, also exhibit high ambient stability in many cases (*e.g.*, BiOI, and most VTPs and double perovskites), with no phase impurities forming after weeks of storage in air. Out of this wide variety of materials, we especially highlight VTPs, which have achieved both the lowest detection limits and highest sensitivities reported in Table 2. Both MA_3_Bi_2_I_9_ and Cs_3_Bi_2_Br_6_ exhibit LoDDs < 1 nGy_air_ s^−1^, along with sensitivities exceeding 10^3^ μC Gy_air_^−1^ cm^−2^. Sensitivities as high as > 10^5^ μC Gy_air_^−1^ cm^−2^ were also reported from 2D Cs_3_Bi_2_Br_6_, and this was likely enhanced through photoconductive gain. Other notable materials include passivated Bi_x_I_y_ and BiOI, which also have low LoDD and high sensitivities. BiOI is particularly promising because of its absence of exciton formation at room temperature (unlike VTPs) or carrier localization (unlike Cs_2_AgBiBr_6_ elpasolites). We therefore believe that these materials are especially worth emphasis in future efforts at developing X-ray detectors from Bi-based materials.

There has been thriving research in the area of Bi-based compounds for X-ray detection. Taking these materials forward, it is important to go beyond simple demonstrations of Bi-based materials in single test devices at the lab scale. For practical use in medical imaging, large-area multi-pixel flat-panel detectors are required. Here, it is important to increase the size of the devices from the mm-level (as achievable with single crystals) to the cm-level, such that these devices can be tiled to form flat-panel imagers. Promising routes forward include the fabrication of polycrystalline wafers (by pressing together single crystals or powders), deposition of thick films (> 200 μm), or the formation of flexible nanocomposite arrays. These materials have the advantage of being more cost-effective to synthesize than large single crystals, but it will be essential to mitigate any increases in dark current or ion migration via grain boundaries or structural defects. This includes performing in-depth characterization to understand the role of grain boundaries on non-radiative recombination and charge-carrier scattering, for example through cathodoluminescence mapping, or fabricating the materials into thin film transistors and measuring the temperature-dependence of the field-effect mobility.^113^ Strategies to address deleterious effects at grain boundaries, surfaces and interfaces include developing passivation approaches and post-deposition heat treatments to reduce the density of structural defects or strain in the materials, such that mobility-lifetime products approach the values of their single-crystal counterparts. Heteroepitaxial passivation has arisen as a particularly promising route. For example, a heteroepitaxial layer of BiOBr was grown onto polycrystalline Cs_2_AgBiBr_6_ to suppress non-radiative recombination and ion migration, such that these materials performed comparably to single crystals in X-ray detectors.^108^ Beyond this, there are a wide range of successful passivation strategies that have been implemented with lead halide perovskites, including using ligands with functional groups that coordinate to surface defects,^114^ doping with alkali halides,^115^ or through physisorption of O_2_/H_2_O species on the surface.^116^ Such strategies could act as inspiration for the development of approaches to passivate surfaces and interfaces in Bi-based materials.

Furthermore, more efforts are needed to integrate these materials with ASICs to develop imagers. A simple approach is to separately grow the wafer or single crystals, and electrically integrate these with the ASIC via bonding techniques. Current approaches include wire bonding, flip-chip bonding, and isotropic conductive film bonding. A critical challenge is that the spatial resolution will depend on the size of the pixels used. For example, 83.2 μm sized pixels on a CMOS with CsPbBr_3_ integrated onto it had a spatial resolution of 5.0 lp mm^−1^.^117^ This is close to the requirement for radiography, but below the requirement for mammography (10 lp mm^−1^). Another challenge is that binding the X-ray attenuating medium and the ASIC can potentially damage the soft Bi-based materials, since this often involves the application of pressure or heating. Such a challenge could be overcome by directly depositing the X-ray attenuating medium onto the ASIC, for example, through thick film deposition from the vapor phase or from solution. However, in this latter approach, the processing temperature of each layer needs to be kept typically below 125°C to avoid damaging the ASIC.

Beyond these rigid device applications, there are also future opportunities for flexible and wearable devices. Here, it is particularly advantageous to have the Bi-based materials in nanocrystal form and integrated into a flexible polymer matrix. When using X-ray detectors in portable and wearable applications, it is a significant advantage if these devices are self-powered, obviating the need for a bulky energy storage device with a limited lifetime. Self-powered operation has been reported in Bi-based materials, for example, through the formation of ferroelectric domains when using chiral molecules within the structure.^90^ Other means of achieving self-powered operation could also be possible, for example, by having a large built-in field engineered via the device interfaces, or by making use of ion migration to form a built-in field in these devices.

Finally, beyond these practical and device-related challenges, it is also important to address key fundamental challenges with these materials. One of the most important barrier is carrier localization, which has been widely found among Bi-based perovskite-inspired materials, and which severely restricts mobility-lifetime products. Although important progress has been made in identifying the chemical and structural factors that allow carrier localization to be overcome, these proposed design principles need to be tested and applied to develop compounds that could achieve delocalized free carriers. But, as found in the case of BiOI, even if band-like transport is achieved, non-radiative losses can still occur as a result of electron–phonon coupling. Furthermore, defects could also induce extrinsic self-trapping, and these effects need to be understood more in these materials.

## Data Availability

No new data was generated.
